# Social Determinants of Health in Cerebral Palsy

**DOI:** 10.3390/jcm13237081

**Published:** 2024-11-23

**Authors:** Salathiel R. Kendrick-Allwood, Melissa M. Murphy, Katie S. Shin, Anmol Minaz, Laverne Keecia Walker, Nathalie L. Maitre

**Affiliations:** 1 Department of Pediatrics, Division of Neonatology, Emory University School of Medicine, Atlanta, GA 30322, USA; salathiel.r.kendrick-allwood@emory.edu (S.R.K.-A.); melissa.murphy@emory.edu (M.M.M.); katie.shin@emory.edu (K.S.S.); laverne.keecia.walker@emory.edu (L.K.W.); 2 Children’s Healthcare of Atlanta, Atlanta, GA 30329, USA; 3 Department of Community Health Sciences, Aga Khan University, Karachi 74800, Pakistan; anmol.minaz@aku.edu

**Keywords:** cerebral palsy, social determinants of health, trauma, depression, adverse childhood experiences, infants, caregivers

## Abstract

**Background/Objectives**: To describe social and psychological needs, such as poverty, early trauma, or adverse childhood events, of caregivers with a child newly diagnosed with cerebral palsy (CP) or receiving a designation of high-risk for cerebral palsy (HRCP). **Methods**: Caregiver self-report questionnaires screening for unmet social needs, adverse childhood experiences (ACEs), depression symptoms, and trauma were collected from 97 caregivers of children with CP/HRCP seen in a high-risk infant follow-up clinic (adjusted age range 1–24 months). We compared their responses to those of 97 caregivers of age-matched controls seen in the same clinic with similar risk factors over the equivalent time period. **Results**: Income insecurity and positive screening rate for depressive and trauma symptoms were high for both groups (CP/HRCP, matched control group); no differences were found between CP/HRCP and control groups. Rates of food and housing insecurity and caregiver ACEs were not different between groups. All families received referrals to appropriate community support at the visit. **Conclusions:** Caregivers of children with CP/HRCP in high-risk infant follow-up clinics may face difficult conversations and decision-making in the context of high psychological and social adversity. Comprehensive support should be considered as early as possible.

## 1. Introduction

Multiple family and caregiver factors may affect the long-term health and resilience of children with developmental disorders, including family social determinants of health (SDH) [1,2], caregiver experiences during childhood [3], and caregiver mental health [4,5,6]. Cerebral palsy (CP), the most common motor disability in children, is an umbrella term used to describe a group of permanent disorders of the development of movement and posture, causing activity limitation [7]. Moreover, it is a developmental disorder in which parental social and psychological factors have been implicated in child outcomes, including condition severity, co-morbidities, and degree of cognitive impairment [1]. National efforts to implement early detection of CP in the US have lowered the age at which a diagnosis of CP is made [8,9]. However, less is known about the familial social and psychological contexts in which these early diagnostic conversations occur, and the potential effects on both child and family outcomes. The present paper describes the social, childhood, and psychological characteristics of parents of infants who are diagnosed with CP or high-risk of CP (HRCP) designation and age-matched peers without CP/HRCP seen in the same high-risk infant follow up clinic in the US.

The term “SDH” encompasses the environments where people are born, live, learn, work, play, worship, and age that affect a wide range of health, functioning, and quality-of-life outcomes and risks. They include economic stability, education, neighborhoods, and built environments, social and community contexts, and healthcare access and quality. Unmet social needs, such as difficulty obtaining food or housing, are increasingly recognized as a critical component of social risk (i.e., food insecurity) associated with from adverse SDH [10]. Health disparities arising from elevated social risk are often experienced by marginalized populations [11]. As it relates to CP, SDH factors such as income, maternal education, and physical environment are associated with child outcomes, including participation in activities and severity of gross motor and bimanual function, see [12] for a review. Consequently, understanding family SDH can help providers identify unmet social needs and resilience factors from which to support families [1,2]. 

In addition to the contributions of SDH to the caregiver and child outcomes, caregiver experiences during their own childhood have short- and long-term consequences for both their own physical, behavioral, and mental health [13] and that of their children [3]. The term adverse childhood experience (ACEs) refers to “harmful or distressing childhood events” [3], p. 146 that take place during childhood and include abuse, neglect, familial mental health (e.g., substance abuse, mental illness), loss of a parent, and witnessing abuse/violence [13]. A recent systematic review by Arnold and colleagues (2023) suggests that, in the general population, the number and type of caregiver ACEs are negatively related to child health, well-being, and developmental outcomes. Thus, in diagnostic conversations related to CP, awareness of potential caregiver resilience and risk factors, such as the number and type of ACEs, can inform the provider with an understanding of the family context and circumstances in which a diagnosis will be received; thereby, enabling service providers to better support caregivers.

The context of psychological health for many families of children with CP is further complicated in high-risk infant follow-up (HRIF) programs, as these families have frequently experienced perinatal trauma [14], with persistent psychologic distress well after the critical medical crisis has passed, or the child is discharged from the intensive care unit [15]. Moreover, mothers of infants born very preterm experience higher depression and anxiety rates across childhood than mothers of full-term infants [16]. These findings support the notion that caregivers of a child with CP may experience high degrees of depression and stress-related symptoms, especially when compounded by co-occurring unmet social needs.

Within HRIF, the degree to which caregiver social and psychological needs are met is associated with historical, current, and structural inequalities in the distribution of social, political, environmental, and economic resources. Indeed, these multiple and interacting factors, including poverty, education inequality, environmental threats, and inadequate access to healthcare, increase the likelihood of caregivers experiencing unmet social and psychological needs. For example, a mother’s psychosocial circumstances and poverty can result in chronic distress during pregnancy, slowing fetal growth rate and increasing the risk of preterm birth [3]. Babies born prematurely already have a greater incidence of failure to thrive, developmental issues, and an increased risk of dying in the first year after birth [3].

Moreover, in a self-perpetuating cycle, unmet caregiver social, and psychological needs can contribute to worsening long-term outcomes of children with neurodevelopmental disabilities, amplifying health disparities and common precursors of CP (e.g., preterm birth, perinatal birth events) [17]. When combined with health disparities and their precursors, CP can also affect parental quality of life [18], perceived spousal support [19], and parental mental health [1]. As such, unmet social and mental health needs of caregivers may amplify health disparities and common precursors of CP (e.g., preterm birth, perinatal birth events) [20,21], and contribute to worsening long-term outcomes of children with developmental disorders, including CP. 

Within the International Classification of Function of Health in Disability framework [8], personal, social and environmental factors represent the “context” in which CP is experienced. As such, they are critical to understanding the other aspects of health and function that make up the whole person, such as family and community participation and functional activities [22]. Addressing caregiver social and mental health needs early maybe one valuable pathway to reduce health disparities, thereby altering the child’s context and improving child outcomes. 

In HRIF, early detection of CP has only been implemented in the US since 2017 [8] and a “high-risk for CP” designation since 2021 [7,23]. Thus, largely unexplored is the specific social and psychological context in which these early detection conversations may occur and the potential effects on both child and family outcomes. As implementation of the early detection of CP began in a regional Georgia HRIF clinic, with a catchment area encompassing seven million people from a predominantly Black and underserved population, a historical precedent was set for quality improvement work in this field. It also afforded the unique opportunity to study parental psychological factors and SDH in families of children at their first counseling visit about CP or HRCP in the first two years. Thus, the purpose of the present paper is to describe the burden of psychological and SDH factors of caregivers of infants with/without CP seen in this large HRIF program. 

While the study’s main goal was purely descriptive, we hypothesized that families of children receiving initial counseling CP/HRCP may already experience a greater burden of unmet social and psychological needs than those of other HRIF children, potentially complicating shared decision-making surrounding CP and later resilience.

## 2. Materials and Methods

### 2.1. Setting

The HRIF clinic in the southeast United States was part of a regionalized system covering the largest, northernmost area of the state and a catchment area of ~7 million inhabitants, urban and rural. In 2021, an early detection program for CP was implemented in the clinic with support from the Cerebral Palsy Foundation. As part of early detection implementation and the clinic’s commitment to a family centered care model, routine screening was implemented for psychological and social needs. Caregivers are provided with the screening forms at the start of the visit. The medical provider reports any concerns from the visit in SBAR format to the social worker. A social worker then reviews the completed forms and meets with any family whose responses indicate unmet psychological or social needs to provide targeted resources and support. If the social worker is not able to meet with the family during the visit, an outreach phone call is made following the visit to ensure the family is provided with needed resources. 

### 2.2. Design

We conducted a prospective study of all cases seen over a 12-month period who were counseled for a diagnosis of CP or designation of HRCP. We compared them to matched controls seen over the same period. Clinic inclusion criteria were like other US HRIF programs (Supplement S1).

This study was approved by the Institutional Review Board of Emory University. Written informed consent by the patients/caregivers was waived due to IRB determination of quality improvement. 

### 2.3. Participants 

*CP/HRCP.* The study included all children, and their caregivers, seen in the clinic between 21 November 2021 and 31 October 2022, and received a diagnosis of CP or HRCP designation (Supplement S2) during the study period (n = 97). The diagnostic conversation was initiated by experienced providers trained through a standardized workshop (https://cpearlydetection.org, accessed on 25 October 2024) and supported in improvement through ongoing feedback by the clinic social worker, present at these conversations. 

To have a CP diagnosis in the clinic, a child must have a clinical history consistent with a perinatal insult, neuroimaging confirmation, genetics work-up if neuroimaging is non-conclusive, motor function test showing activity limitations, neurological exam consistent with CP and Hammersmith Infant Neurological Exam scores below cut-off for age, or with asymmetry scores after 9 months, e.g., [24]. The General Movements Assessment (GMA) at 3–4 months corrected age was used as a supporting element, but not a diagnostic assessment [25]. (Supplement S2 contains the standardized checklist for assigning HRCP designations). 

*Matched controls*. Infants seen during the same 12-month time period, but who did not meet diagnostic criteria for CP/HRCP, were matched pairwise to infants with CP/HRCP within one month of the infant’s birthdate and clinic visit date in which the diagnosis or designation was given occurred. If more than one child met the age match criteria, the selection was made to match the infant’s sex. This approach led to selection of 97 matches from the 869 infants under two years seen in the HRIF clinic during the study period with similar risk factors for being seen in the clinic (see Table 1). 

### 2.4. Data Collection and Instruments

Data were collected for the visit at which diagnosis of CP or classification of HRCP occurred (CP/HRCP) or matched clinic visit (control). Upon completion of the questionnaires, the clinic social worker reviewed answers with the caregiver and provided all necessary support based on a directory of community resources. 

#### 2.4.1. Social Determinants of Health

*Family Resource Survey* (FRS). At the time of our study, this caregiver-report survey was in use within the general pediatric practice clinics at our institution. It includes 17 multiple-choice items derived from the original FRS, a tool published by the UK Department of Works and Pensions [26]. The FRS also collects (1) caregiver demographic information (e.g., education level, relationship status); (2) major housing, income, and food needs; (3) emotional support sources; and (4) current exposure to drugs, domestic violence, or abuse. Additionally, the clinic’s FRS captured caregiver resource needs via an open-ended question asking caregivers to indicate if they wished “help with” a given resource (e.g., transportation, their own education, job placement, daycare, and other necessary resources). The FRS form used in our clinic is provided in Supplement S3.

#### 2.4.2. Psychological Needs 

*Depression screening.* Two items in our clinic’s FRS, derived from the Patient Health Questionnaire-2 (PHQ-2), are validated for use as a depression screener [27]. These items asked about the number of days out of the “last 2 weeks” when the caregiver (1) felt down, depressed, or hopeless, or (2) had almost no interest or pleasure” in things. Cases where caregivers endorsed answer choices of several or more days to either of these questions were followed up by the clinic social work team. 

*Trauma. Primary Care Post-traumatic Stress Disorder Screen for* DSM-5 (*PC-PTSD-5*) is a question set asking about instances of trauma leading to unwanted memories, their duration, and tractability (the form used in the clinic is available in Supplement S3, questions 32–37). The initial question asks, “in the past month, have you had any unwanted memories of (EVENT) while you were awake, so not counting dreams”. Cases where caregivers indicated “yes” or described bothersome/intractable memories were followed up by the clinic social work team. Surveys that were left blank were treated as missing data.

*Adverse Childhood Experiences (ACES).* Two versions of ACE inventories were used during the study period. Kaiser ACEs [28] (n = 72) were used until 1 January 2022 when the clinic switched to Philadelphia ACEs (n = 117) to better account for neighborhood exposures [19]. Both ACE versions ask caregivers to indicate the presence of potentially traumatic events in their own childhood, such as experience of physical, sexual, or psychological abuse, witnessing violence, and living among family with mental health issues, substance abuse, or who were incarcerated. 

An ACE was present when a caregiver indicated “yes” to one of the listed items. Consistent with findings from the Philadelphia ACEs study, we used a threshold of 4 or more ACEs to distinguish those at higher risk for poor health outcomes from those with less risk [28,29]. No difference in the number of caregivers with 4 or more ACEs was found between ACE versions (*p* = 0.77, Table 2) so results from the two inventories were combined. For reference, Table 2 and Table 3 present ACEs combined scores as well as ACEs by version.

### 2.5. Statistical Analysis 

Descriptive statistics were reported as frequency, mean, and standard deviation. Comparative analyses included Pearson’s Chi-square and one-way ANOVAs with group as the between-subjects factor for continuous variables were performed for exploratory purposes using SPSS version 29 [30].

In both the CP/HRCP and matched control groups, mothers made up most of the caregivers attending the visit. Thus, when multiple caregivers (e.g., mother and father) attended, only mothers’ responses were included in the present study. Infants without complete caregiver questionnaires were excluded. 

Due to sample size limits and the complexity of analyses, SDH-related items on the FRS were collapsed into four categories of social need: income, housing, food, and access to care. Cases where caregivers indicated a social need was unmet were followed up by the clinic’s social work team. To determine the overall number of unmet social needs, we counted the number of categories (out of 4) with unmet needs indicated. 

## 3. Results

During the study period, questionnaires were completed for 97 (out of 119) children by eligible caregivers (37 with CP counseling, 60 with HRCP counseling). Of note, 27/60 children converted to a diagnosis of CP within the study period while 25/60 had ongoing neuromotor concerns and 3/60 had not yet had a follow-up. No differences emerged between groups on sex, ethnicity/race, or history of prematurity (Table 1). As expected, Hammersmith Infant Neurological Exam (HINE) scores for the CP/HRCP were lower than controls, F(1,180) = 84.48, *p* < 0.001, confirming the group was distinguishable on a CP-specific criterion. 

### Psychological and SDH Factors

There were no group differences in frequency unmet social or mental health needs (Table 2). 

In the entire caregiver cohort (n = 194), 21% met the initial screening criteria for depressive symptoms. The initial screening for PTSD symptoms was positive for 20% of caregivers. Four or more ACEs were reported by 13% of parents. Examination of individual ACEs indicated that 50% of caregivers reported experiencing discrimination, 68.4% had felt unprotected (feeling unsupported, unloved, and/or unprotected), and 75% had lived with a family member experiencing mental illness (Table 3).

**Figure 1:**
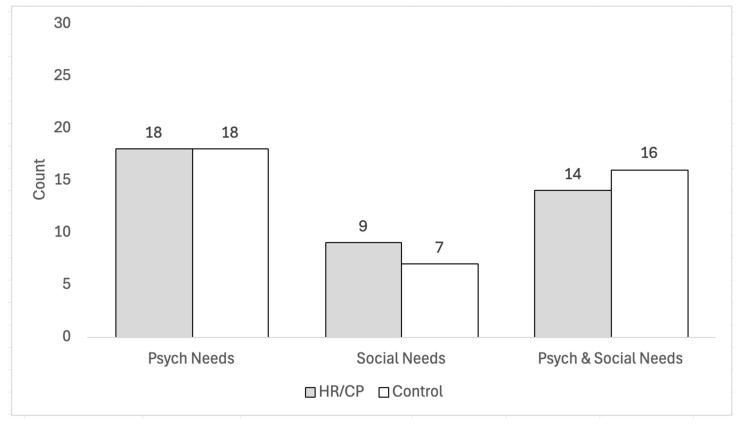
Frequency of co-occurring psychological factors with social needs by group.

In this cohort, we found no difference between groups in the frequency at which unmet social and psychological needs co-occurred. However, we noted that more families with unmet social needs also screened positive for depressive or PTSD symptoms than did not (Figure 1). 

Of note, 32.7% of caregivers did not report unmet social needs when asked standard yes/no questions. Instead, they reported the need when asked to indicate if they wanted help with specific needs (e.g., employment, denial of SSI). This question format also allowed caregivers to report other types of resource needs such as help finding specialized daycare or preschools (n = 24), medical-legal needs (n = 4), parenting concerns (n = 7), finishing their own schooling (n = 5), and faith concerns (n = 1).

## 4. Discussion

This study describes the social and psychological needs of caregivers of an infant with a CP diagnosis or HRCP designation at the time of diagnosis in an HRIF clinic relative to age-matched controls also seen in the clinic. We hypothesized that families of children who were receiving a new diagnosis of CP or being counseled about HRCP may have a greater burden of unmet social and psychological needs than other HRIF children. Contrary to expectations; however, the social and psychological needs of families of a child with CP/HRCP were comparable to those of other infants seen in this HRIF program. Thus, both groups experience a high burden of unmet social and psychological needs that may influence outcomes over the lifespan, including resilience. 

To better understand the degree of social and psychological burden of these families, we explored how rates in our sample compare to the general US population. Rates of income insecurity in our sample were higher than the 11.5% reported in the general population [31]. The difference in income insecurity in our sample was especially pronounced for Black/African American caregivers, among whom 20% reported that their income did not meet their family’s basic needs. 

In contrast, food insecurity reported among the clinic population was comparable to the rates reported in the US population at 8.8% [32], potentially reflecting access to federally funded programs like Women, Infant, and Children’s (WIC) and Supplemental Nutrition Assistance Program (SNAP). Regarding housing insecurity, the lack of a common housing report makes it difficult to define and determine comparable rates to our sample in the general population. Although not statistically significant, 9% of Black/African American caregivers compared to 2% of White caregivers in our sample report being at risk of losing housing, unhealthy conditions at home, or current homelessness. A recent study examining the lack of follow up among over 19,000 infants in a US sample from the Vermont Oxford Network found that relative to White infants, Black and Hispanic infants were overall at lower odds of being seen in high-risk infant follow up [33]. Taken together, it is possible that, especially for Black/African American families, other factors, such as the child’s medical complexity, other caregiving factors (e.g., transportation, childcare), and structural racism present barriers to accessing the HRIF clinic for families who are at the greatest risk of having unmet social needs. 

In the context of early onset disability, cumulative disproportionate negative effects of structural racism are a probable cause of poor obstetrical outcomes faced by Black women in the US, with continued intergenerational discrimination and disinvestment [34]. Consistent with this notion, a systematic review of racial and ethnic disparities in NICU care suggests health and social disparities both influence the likelihood of pre-term birth and reflect racial and ethnic differences in the quality of and access to care [35]. Therefore, to maximize the hypothesized benefits of screening for CP and related developmental disorders, it is imperative to understand the barriers families encounter in processing the information HRIFs provide and accessing resources to which they have been referred. Screening for caregiver ACEs on its own can help providers understand the context in which they counsel families and offer support. Screening for unmet social needs and resource referrals may help break a vicious cycle for infants with developmental risks: unmet social needs contribute to early neural insults and poor intrauterine brain development, which can then be compounded by deprivation or alterations of the postnatal environment and familial context. For families whose children are diagnosed early with CP, early health disparities are then augmented by inequalities associated with their child’s disability [1].

Regarding psychological needs, screening for PTSD symptoms and depressive symptoms (see Table 2) suggest potential vulnerabilities in our sample that require additional assessment. If accurate, screening rates in our sample suggest that the rates of unmet psychological needs may exceed that of the US adult population (3.6% PTSD, 8.3% depression) [36]. As with income insecurity, these differences were especially pronounced for Black/African American caregivers, among whom 26% screened positive for possible depressive symptoms and 22% were positive for possible PTSD symptoms. When these elevated rates of psychological concerns co-exist with poverty and unmet social needs, chronic distress can result in worsening outcomes and perpetuating health disparities. The high rates of risk for mental health concerns observed in our sample suggest that trauma-informed care is a necessary addition to counseling families of children in HRIF care to address caregiver mental health [37]. While screening rates were not increased in families in our clinic during the first conversations surrounding CP, they were still high and should be considered for their future implications. Parents of children with CP may be at particular risk for experiencing a higher burden of stress, anxiety, and depression related to their child’s function, chronic pain, and participation. Therefore, careful surveillance of parental mental health and provision of treatment at the time of early CP diagnosis may be an important complement to typical screening for behavioral concerns.

The present study highlights the high burden of unmet social and psychological factors in the first years after birth among families of a child in HRIF. However, it is important to note that sample size may limit the detection of small effects. Additionally, the self-report questionnaire format at times led to missing or conflicting data. First, almost 30% of participants did not show immediate income insecurity on the multiple-choice questions but did note income-related concerns on the “do you need help with …” questions (e.g., help with own schooling, SSI denial, or employment; see Supplement S4). One explanation for this apparently conflicting response pattern is that the absence of insecurity is not synonymous with security (i.e., that assistance is not needed with employment or supplemental income). To better understand resource use by these families, additional work by our team explores rates of resource access and utilization (manuscript in preparation).

Second, at times caregivers who did not meet positive screening criteria on the PTSD screening questionnaire would go on to describe symptoms of consistent PTSD to the social worker during in-clinic questionnaire review. These anecdotal reports suggest that the lower rates of positive PTSD screenings may be an underestimation of the true need for additional PTSD assessment and resources. Fathers were noted in several instances to have mothers complete the PTSD screening questions despite being willing to fill out the other questionnaires. As such, additional investigation into specific psychological needs of fathers is essential. Given that the results presented here focused on mothers as primary caregivers, the generalizability to fathers is unknown. 

Fourth, the switch from Kaiser to Philadelphia ACEs may contribute to under reporting of discrimination, time in foster care, and witnessing violence since these items were not included in the Kaiser version [28]. Finally, while we did not find differences in unmet social and psychological needs between CP/HRCP and matched controls at the initial conversation surrounding the disorder, differences may emerge in subsequent years as CP/HRCP-related symptoms emerge (e.g., function, chronic pain, mobility, and participation) [38,39].

Fifth, it is noteworthy that the children included in the present study were young (adjusted age at visit < 24 months). It is possible as the children age, changes may occur in the psychological and/or social needs of these caregivers and families. Although following these families over time was beyond the scope of the present study; longitudinal comparisons of individual and group trajectories through the pre-school and into the school-age years are needed.

Regardless, the present study highlights that challenging conversations of new CP diagnoses or HRCP designation occur in potentially charged contexts. Underreported financial insecurity, access needs, and other barriers to care, along with mental health concerns, potentially complicate shared decision-making surrounding CP, and later resilience. Our data likely underestimate the actual burden of social and psychological factors as they are not always disclosed until a social work professional is involved. As such, current data strongly supports the need for systematic screening of high-risk infants, and access to social workers and psychologists in HRIF and early CP programs. Our findings suggest patients cared for in both early CP detection programs and NICU follow-up clinics would benefit from a wraparound care approach that includes routine screening for psychological and social needs among caregivers complemented by social work involvement to connect families with resources.

Once identified, to harness a child’s potential, a “social prescribing scheme” (provision of non-medical prescriptions by healthcare providers, to improve the health and well-being and to strengthen community connections) might be adopted in response [40]. This approach may be particularly appropriate for caregivers/families of children with CP or HRCP designation to promote access to early family centered care services within the context of the ICF rather than only addressing the medical needs inherent to CP [41].

## Figures and Tables

**Table 1:** Cohort Characteristics.

Characteristics	CP/HRCP (n = 97)	Controls (n = 97)	Statistic
Sex (n, % male)	54 (56%)	54 (56%)	X^2^(1) = 0.00, *p* = 1.0
Ethnicity (n, % Non-Hispanic)	89 (92%)	92 (95%)	X^2^(1) = 0.74, *p* = 0.57
Race (n, %)			X^2^(1) = 4.21, *p* = 0.38
Black American	60 (62%)	55 (57%)	
White	26 (27%)	35 (36%)	
Other	11 (11%)	7 (7%)	
Chronological age at visit (in months): M(SD)	10.02 (5.45)	10.44 (5.01)	-
Adjusted age at visit (in months): M(SD)	7.61 (5.11)	8.19 (5.29)	-
Gestational Age at birth (in weeks): M(SD)	29.4 (5.69)	30.22 (4.59)	-
History of prematurity (n, % yes)	78 (80.41)	83 (85.56)	X^2^(1) = 0.91, *p* = 0.45
Chief complaint at initial visit other than prematurity (n, %)			
HIE	15 (15%)	8 (8%)	
Intracranial Abnormalities	14 (14%)	4 (4%)	
Low Birthweight	6 (6%)	8 (8%)	
Seizure	11 (11%)	1 (1%)	
Multiple Gestation	2 (2%)	6 (6%)	
Pulmonary Hypertension	0 (0%)	2 (2%)	
Other	5 (5%)	6 (6%)	
HINE Total score: M(SD)	52.08 (10.95)	66.36 (9.81)	F(1,179) = 84.68, *p* < 0.001
HINE Asymmetry: M(SD)	3.60 (2.86)	1.78 (1.78)	F(1,158) = 21.66, *p* < 0.001

**Table 2:** Frequency of caregiver social and psychological needs by group.

	CP/HRCP (n = 97) ^a^	Controls (n = 97) ^a^	Total (n = 194)	Statistic
Depressive screener positive (n, %)	16 (17%)	25 (26%)	41 (21%)	X^2^(1) = 2.51, *p* = 0.16
PTSD screener positive (n, % yes)	17 (22%)	15 (19%)	32 (21%)	X^2^(1) = 0.31, *p* = 0.69
	(n = 76)	(n = 80)	(n = 156)	
Adverse Childhood Experiences ≥ 4 (n, %)	10 (13%)	9 (10.47)	19 (12%)	X^2^(1) = 0.25, *p* = 0.63
	(n = 77)	(n = 86)	(n = 163)	
Kaiser At least 1 ACE	(n = 41)	(n = 31)		X^2^(1) = 0.1, *p* = 1.0
	34 (83%)	26 (84%)	-	
≥4 ACEs	1 (2%)	4 (13%)		
Philadelphia At least 1 ACE	(n = 53)	(n = 64)		X^2^(1) = 0.4, *p* = 0.85
	30 (57%)	35 (54%)	-	
≥4 ACEs	9 (17%)	5 (8%)		
Unmet social needs (n, %)				
Income	19 (20%)	13 (14%)	32 (17%)	X^2^(1) = 1.51, *p* = 0.25
	(n = 94)	(n = 96)	(n = 190)	
Housing	6 (6%)	5 (5%)	11 (6%)	X^2^(1) = 0.10, *p* = 1.0
Food	3 (3%)	6 (6%)	9 (5%)	X^2^(1) = 1.05, *p* = 0.50
Access to care	5 (5%)	6 (6%)	11 (6%)	X^2^(1) = 0.12, *p* = 1.0
One or more unmet social need reported (n, % yes)	27 (28%)	25 (26%)		X^2^(1) = 0.11, *p* = 0.87
Parent informational needs (n, %) ^b^				
Finding daycare	8 (8%)	11 (11%)		-
Finding preschool	2 (2%)	1 (1%)		
Child’s school/individual education plan (IEP)	0 (-)	0 (-)	0 (-)	-
Medical equipment	4 (4%)	3 (3%)		-
My own schooling	2 (2%)	2 (2%)		-
Child custody issues	2 (2%)	1 (1%)		-
Faith concerns	0 (-)	0 (-)	0 (-)	-
Parenting issues/support	2 (2%)	4 (4%)		-

^a^ Except when otherwise indicated due to caregiver non-response; ^b^ percentages are likely underestimates due to ambiguous non-responses, which were treated as missing data. Note. CP, Cerebral Palsy; HRCP, High Risk for CP; “Income” included need for employment, job training, or denial of public benefits/social security); “housing” included lack of housing, unsafe, or unhealthy housing; “food” included lack of food or worry that food would run out; “access” included lack of transportation, medical equipment, or service providers in their region; depressive symptom screener and social factors were determined based upon the Family Resource Survey (FRS).

**Table 3:** Frequency of Adverse Childhood Experiences (ACEs) Type by Group: n (%) **^a^**.

ACE Version	ACE Type	CP/HRCP (n = 77)	Controls (n = 85)
Kaiser & Philadelphia	Lack of food, clothing, home, protection	2 (3%)	2 (2%)
	Sexual Abuse	3 (4%)	4 (5%)
	Jail	4 (5%)	8 (10%) ^d^
	Mental Abuse	5 (6%)	8 (9%)
	Physical Abuse	5 (7%) ^c^	8 (9%)
	Violent Harm	6 (8%)	7 (8%)
	Drugs	8 (10%)	8 (9%)
	Mental Illness	8 (10%)	9 (11%)
	Feelings of being unprotected	7 (9%)	10 (12%)
	Divorce	29 (38%)	29 (34%)
Philadelphia only	Witness violence ^b^	2 (2%) ^c^	1 (1%)
	Experience in Foster Care ^b^	2 (3%)	4 (5%)
	Discrimination ^b^	15 (20%)	14 (17%)

**^a^** Actual total sample sizes across types vary between 88 and 121 due to ambiguous non-responses and inclusion of 3 additional items on the Philadelphia ACEs version, so both type and group n are reported. Therefore, rates are likely to be underestimates. ^b^ Item was included on Philadelphia ACEs but not the Kaiser ACEs version. ^c^ based on n = 76. ^d^ based on n = 84. Note. CP, Cerebral Palsy; HRCP, High Risk for CP.

## Data Availability

The raw data supporting the conclusions of this article will be made available by the authors upon reasonable request.

## Supplementary Materials

The following supporting information can be downloaded at: https://www.mdpi.com/article/10.3390/jcm13237081/s1, Supplement S1: High-Risk Infant Follow-up (HRIF) Criteria; Supplement S2: High-Risk for Cerebral Palsy (HRCP) Designation Criteria; Supplement S3: Clinic Questionnaires; Supplement S4: Differences in reporting social determinants of health based upon question format.

## Abbreviations

ACES, Adverse Childhood Experiences; CP, Cerebral Palsy; HINE, Hammersmith Infant Neurological Exam; HRCP, High Risk for Cerebral Palsy; HRIF, High-Risk Infant Follow-up; SDH, Social Determinants of Health.
