# Supplementary material for: Social Determinants of Health in Cerebral Palsy
Source: J Clin Med. 2024 Nov 23;13(23):7081. doi: 10.3390/jcm13237081 (PMC11642413; doi:10.3390/jcm13237081)
Supplement: Supplementary file 1 [file jcm-13-07081-s001.zip › Kendrick-Allwood_Supplement 1_HRIF Criteria.pdf]

Supplemental materials

Supplement S1: Criteria for Participation in High-Risk Infant Follow up Program

1. Preterm infant with birth weight <1500 gms or </= 32 weeks gestational age at birth*
2. Hypoxic Ischemic Encephalopathy or Neonatal Encephalopathy (mild, moderate or severe)*
3. Meningitis, encephalitis or other infectious brain insult (congenital or postnatal)*
4. Neonatal seizures*
5. General Movements Assessment: Cramped Synchronized or Absent Fidgety*
6. Congenital diaphragmatic hernia
7. Severe persistent neonatal hypoglycemia with documented neurological concern
8. Microcephaly (HC<5%) or any brain insult/congenital abnormality on neuroimaging *
9. Hyperbilirubinemia requiring exchange transfusion
10. ECMO
11. Multiple gestation if at least one infant meets criteria
12. Other: Community Pediatrician* or Neonatologist reason for neurodevelopmental referral – for community referrals, only under 36 months corrected age

**Denotes criteria* that may constitute newborn or infant attributable risks per Novack (2017)
