# Supplementary material for: Social Determinants of Health in Cerebral Palsy
Source: J Clin Med. 2024 Nov 23;13(23):7081. doi: 10.3390/jcm13237081 (PMC11642413; doi:10.3390/jcm13237081)
Supplement: Supplementary file 1 [file jcm-13-07081-s001.zip › Kendrick-Allwood_Supplement 2_HRCP designation.pdf]

High Risk for Cerebral Palsy Designation Checklist

Many elements are involved in the diagnosis of cerebral palsy (CP) and high risk for CP (HRCP) designation. This checklist may help with the decision to use the term HRCP, or choose a follow-up per your clinic's protocols.

SITUATION 1 – INFANT WITH NEWBORN ATTRIBUTABLE RISKS

Consider diagnosis of cerebral palsy (CP) if 6 criteria present (including clinical history)
Consider high risk for cerebral palsy (HRCP) designation if 4 criteria + clinical history present (missing 1 diagnostic element)
Note MRI <u>OR</u> positive genetic testing for condition count as a single criterion

- [x] **Clinical history consistent**
  e.g. prematurity, fetal growth restriction, birth asphyxia, intrauterine drug exposure
- [ ] **Neurological exam consistent**
  e.g. hypertonia, dystonia, head lag, absent parachute reflex in infant >12 months, hyperreflexia
- [ ] **Motor function impaired**
  e.g. clinical therapist impression, tests (e.g. TIMP, AIMS, NSMDA) showing impairment
- [ ] **Neuroimaging**
  e.g. brain MRI or ultrasound with findings consistent with hypoxic-ischemic encephalopathy, grade 3-4 intraventricular hemorrhage, hydrocephalus, stroke, periventricular leukomalacia, sequelae of infection
- [ ] **<u>OR</u> if MRI unremarkable then genetic testing consistent**
  e.g. Lesch-Nyhan syndrome
- [ ] **GMA**
  e.g. cramped synchronized (suggestive of spastic CP), absent fidgety
- [ ] **HINE scores consistent**
  e.g. Total score for age below expected cut-offs, asymmetry score age 9 months

SITUATION 2 – INFANT WITH ONLY INFANT ATTRIBUTABLE RISKS/FIRST EVALUATION

Consider HRCP designation if 4 criteria present (missing 1 diagnostic element)
Note MRI <u>OR</u> positive genetic testing for condition count as a single criterion

- [ ] **Neurological exam consistent**
  e.g. hypertonia, dystonia, head lag, absent parachute reflex in infant >12 months, hyperreflexia
- [ ] **Motor function impaired**
  e.g. clinical therapist impression, tests (e.g. TIMP, AIMS, NSMDA) showing impairment
- [ ] **HINE scores consistent**
  e.g. Total score for age below expected cut-offs, asymmetry score age 9 months
- [ ] **Neuroimaging**
  e.g. brain MRI or ultrasound with findings consistent with hypoxic-ischemic encephalopathy, grade 3-4 intraventricular hemorrhage, hydrocephalus, stroke, periventricular leukomalacia, sequelae of infection
- [ ] **<u>OR</u> if MRI unremarkable then genetic testing consistent**
  e.g. Lesch-Nyhan syndrome
- [ ] **GMA**
  e.g. cramped synchronized (suggestive of spastic CP), absent fidgety

SITUATION 3 – CONVERTING FROM HRCP TO CP

Consider conversion to CP diagnosis if all 4 criteria present

- [ ] **Repeat Neurological Examination consistent between visits**
  e.g. hypertonia, dystonia, persistent head lag, absent parachute reflex in infant >12 months, hyperreflexia
- [ ] **Neuroimaging**
  e.g. brain MRI or ultrasound with findings consistent with hypoxic-ischemic encephalopathy, grade 3-4 intraventricular hemorrhage, hydrocephalus, stroke, periventricular leukomalacia, sequelae of infection
- [ ] **Motor function impaired – not only delayed**
  e.g. clinical therapist impression, TIMP, AIMS, NSMDA – this is important in the case of preterm infants whose motor delays may resolve and in the case of children with CP who may walk by age 2 but with functional impairments
- [ ] **Repeat HINE scores consistently below cut-offs**
  e.g. Total score for age below expected cut-offs, asymmetry score age 9 months

Test of Infant Motor Performance (TIMP), Alberta Infant Motor Scale (AIMS), Neuro-sensory Motor Developmental Assessment (NSMDA).
This document was created by Dr F. Kim MD and Professor N. Maitre MD,PhD for the Cerebral Palsy Foundation based on the published consensus statement in J. Pediatric Rehabilitation Medicine (2022)

Test of Infant Motor Performance (TIMP), Alberta Infant Motor Scale (AIMS), Neuro-sensory Motor Developmental Assessment (NSMDA).
This document was created by Dr F. Kim MD and Professor N. Maitre MD,PhD for the Cerebral Palsy Foundation based on the published consensus statement in J. Pediatric Rehabilitation Medicine (2022)
