# Supplementary material for: Social Determinants of Health in Cerebral Palsy
Source: J Clin Med. 2024 Nov 23;13(23):7081. doi: 10.3390/jcm13237081 (PMC11642413; doi:10.3390/jcm13237081)
Supplement: Supplementary file 1 [file jcm-13-07081-s001.zip › Kendrick-Allwood_Supplement 3_DPC-FRS.pdf]

Supplement S3: Family Resource Survey (FRS)

Based on a modified version of a published UK Survey by the Department of Works and Pensions: Family Resources Survey 2018/19: Background note and methodology (publishing.service.gov.uk).

Children's Healthcare of Atlanta

EMORY UNIVERSITY

DEVELOPMENTAL PROGRESS CLINIC

Name ______________________________

Date of Birth ________________________

MRN#_______________________________

This clinic is dedicated to providing the best possible care for your child. In order for us to better serve you, please take a few minutes to answer the following questions. Your answers will be kept strictly CONFIDENTIAL as part of child's medical record. Please circles your answer.

1. Are you the child's legal guardian?

   Yes No

2. Are you the child's: Mother Father Grandparent Foster Parent

   Other relative Other _______________________________________

3. What is your relationship status? Single, not in a relationship Significant relationship (not living together) Live-In Partner Married Divorced

4. What is the highest grade you completed? 1 2 3 4 5 6 7 8 9 10 11 12 High school GED Some college or Vocational school College Grad or Above

5. Does this child live primarily in your home?

   Yes No

6. Does your income support your family's basic needs?

   Yes No

7. Do you have any major housing problems?

   No   Currently homeless   At risk of losing housing   Unhealthy conditions in home

8. Do you worry that your neighborhood is unsafe for your child or family?

   Yes   No

9. Within the past 12 months have you worried that your food would run out before you got money to buy more?

   Yes   No

10. Within the last 12 months did you run out of food before you could get money to buy more?

    Yes   No

11. Where do you get emotional support? (Circle all that apply)

    Family   Friends   Faith or religious group   No support

    Other________________

12. Over the last 2 weeks, how many days have you felt down, depressed, or hopeless?

    A) No days B) Several days C) More than half the days D) Nearly every day

13. Over the last 2 weeks, how many days have you felt <u>almost no interest or pleasure</u> in doing things?

    A) No days B) Several days C) More than half the days D) Nearly every day

14. Do you tend to recover from illnesses or hardship(s)?

    A) Rarely true   C) Sometimes true

    B) Often true   D) True nearly all of the time

15. In the past year has your partner or other family member pushed you, punched, kicked you, or threatened to hurt you?   Yes   No

16. Do you or your partner have a history of alcohol or drug use? (including marijuana, opioids, cocaine, etc).

Yes No

17. Do you worry that your child has been physically or sexually abused?

Yes. No

18. Your parents or guardians were separated or divorced?

A) Yes B) No C) Not Sure D) Not Applicable (N/A)

19. You lived with a household member who served time in jail or prison?

A) Yes B) No C) Not Sure D) Not Applicable (N/A)

20. You lived with a household member who was depressed, mentally ill or attempted suicide?

A) Yes B) No C) Not Sure D) Not Applicable (N/A)

21. You saw or heard household members hurt or threaten to hurt each other?

A) Yes B) No C) Not Sure D) Not Applicable (N/A)

22. A household member swore at, insulted, humiliated, or put you down in a way that scared you OR a household member acted in a way that made you afraid that she/he might physically hurt you.

A) Yes B) No C) Not Sure D) Not Applicable (N/A)

23. Someone touched your private parts or asked you to touch their private parts in a sexual way.

A) Yes B) No C) Not Sure D) Not Applicable (N/A)

24. More than once you went without food, clothing, a place to live, or had no one to protect you.

A) Yes B) No C) Not Sure D) Not Applicable (N/A)

25. Someone pushed, grabbed, slapped or threw something at you OR you were hit so hard that you were injured or hand marks.

A) Yes B) No C) Not Sure D) Not Applicable (N/A)

26. You lived with someone who had a problem with drinking or using drugs?

A) Yes B) No C) Not Sure D) Not Applicable (N/A)

27. You often felt unsupported, unloved and/or unprotected.

A) Yes B) No C) Not Sure D) Not Applicable (N/A)

28. How often do you feel you were treated badly or unfairly because of your skin color, language/accent, or because you come from a different country or culture?

A) Very often B) Often C) Rarely D) Very Rarely E) N/A

29. Were you ever in Foster Care?

A) Yes B) No C) Not Sure

30. How often if ever, did you see or hear a parent, stepparents or another adult helping raise you being slapped, kicked, punched, or beaten up?

A) Many times B) A few times C) Once D) Never

31. Would like help with any of these issues? (Circle all that apply)

A) Finding daycare B) Finding preschool C) Child's school/EP D) Car Seat

E) Medical Equipment F) My own schooling G) Child custody issues

I) Faith concerns J) Denial of SSI or public benefits H) Job Training

K) Transportation L) Employment M) Parenting issues/support

Other ___________________________

32. In the past month have you had any unwanted memories of (EVENT) while you were awake, so not counting dreams?

33. How does it happen that you start remembering (EVENT)?

34. [If not clear:] (*Are these unwanted memories, or are you thinking about [EVENT] on purpose?)*

35. How much do these memories bother you?

36. Are you able to put them out of your mind and think about something else?

37. How often have you had these memories in the past month?
