# Supplementary material for: Social Determinants of Health in Cerebral Palsy
Source: J Clin Med. 2024 Nov 23;13(23):7081. doi: 10.3390/jcm13237081 (PMC11642413; doi:10.3390/jcm13237081)
Supplement: Supplementary file 1 [file jcm-13-07081-s001.zip › Kendrick-Allwood_Supplement 4_Data Table.pdf]

Supplement S4: Differences in reporting social determinants of health base d upon question format

Insecurity Type	Indicated "Yes" on Forced Choice only	Indicated "Yes" on open-ended only	Indicated "Yes" on Both	Total
Income	11	15	6	32
Housing	8	1	2	11
Food	7	1	1	9
Total ≥1	36	17	9	52

Note. Forced yes/no choices were initially asked in the questionnaire. After ascertaining other social determinants of health, open-ended questions were asked as "What help would you like with any of the following?" And "Do you have other needs?"
